# Characterisation of adult patients with neuroendocrine neoplasms and their journey to diagnosis

**DOI:** 10.1038/s44276-025-00198-3

**Published:** 2026-01-13

**Authors:** Busani Ndlela, Ruth Swann, Georgios Lyratzopoulos, Sally Vernon, Brian Rous, Sean McPhail, Greg Rubin

**Affiliations:** 1 National Disease Registration Service, NHS England, Leeds, UK; 2 Cancer Intelligence, Cancer Research UK, London, UK; 3 Epidemiology of Cancer Healthcare and Outcomes (ECHO), Department of Behavioural Science and Health, Institute of Epidemiology and Health Care (IEHC), University College London, London, UK; 4 Population Health Sciences Institute, Newcastle University, Newcastle Upon Tyne, UK

## Abstract

**Background:**

Neuroendocrine neoplasms (NENs) are rare and often present with non-specific symptoms. Diagnostic delays of 4 years or more have been reported in patient surveys, primarily attributed to low recognition of symptoms and multiple primary care consultations. However, population-based evidence is sparse. We characterised diagnostic pathways to guide improvement efforts.

**Methods:**

We used National Cancer Diagnosis Audit (NCDA) 2018 data for all adults diagnosed with NEN of eleven cancer sites. Primary care-led investigations were grouped into blood, urinary, imaging, endoscopy, and other. The number of pre-referral consultations, the primary care interval (PCI), and the diagnostic interval (DI) were measured.

**Results:**

Data were available on 919 adult patients. Case-mix by cancer site was comparable to national cancer registry data. 45% were referred as an Urgent Suspected Cancer; 18% as an emergency. The median PCI and DI were 7 and 54 days, respectively (2 and 36 among NCDA cases overall). Of 633 patients (69%) with at least one recorded GP consultation, 25% (161/633) had three or more pre-referral consultations. 30% of patients underwent diagnostic imaging.

**Conclusions:**

Comparatively, median PCI and DI were longer (and use of diagnostic tests greater) for NEN than other cancers in the NCDA, but substantially shorter than previously reported.

## Background

Neuroendocrine neoplasms (NENs) are a diverse group of relatively rare neoplasms. They can be broadly subdivided into well-differentiated neuroendocrine tumours (NETs) and poorly differentiated neuroendocrine carcinomas (NECs)—see full definition in Appendix 1. Their incidence is increasing globally [1–3] and for England it was 8.6 per 100,000 in 2018 [4], although reported rates depend upon the precise definition of NEN, which can vary between reporting groups. A full-time GP with an average list of 1700 patients can therefore expect to see one new NEN approximately every 7 years. NENs can arise in any organ where neuroendocrine cells are present, but are most commonly found in the lungs and gastrointestinal tract. Symptomatic presentation is highly variable, depending on site and spread, and whether the neoplasm is hormone-secreting.

Long delays of over 4 years in the diagnosis of NENs have been reported, based on patient surveys in the UK and North America [5, 6]. These delays have been primarily attributed to the primary care phase of the diagnostic pathway, due to misdiagnosis and low recognition of symptoms [2, 5–7]. In the UK, these findings have driven calls by cancer charities and patient advocates to improve GP awareness [8] and to develop NEN care pathways [9].

The most recent English National Cancer Diagnosis Audit (NCDA) gathered detailed, clinician-curated information on diagnostic pathways in primary care and referral to secondary care for 64,489 patients diagnosed with cancer in 2018, linked to cancer registration data [10]. This represented approximately 20% of the incident malignant cancers for England in that year, excluding non-melanoma skin cancers [11].

We set out to characterise the diagnostic pathways for patients with NENs reported in the NCDA, to help better understand the nature and size of the problem in a way that can inform the development of optimal NEN care pathways.

## Methods

### Data source

Data from the NHS England National Disease Registration Service (NDRS, the organisation responsible for cancer registration in England) were used [12]. Patients included in this register and registered at practices participating in the 2018 National Cancer Diagnosis Audit (NCDA) [10] formed the cohort for this study. Data were selected for all individuals who had been diagnosed with NEN in 2018, although the design of the NCDA included only malignant tumours (defined according to ICD-10) and excluded NENs of the skin.

The NCDA is a large population-based audit in which more than a quarter of all practices in England participate. Its strengths and weaknesses are more fully described elsewhere [10, 13]. In brief, cases were identified from the English cancer registration system, with detailed information added from the primary care patient record by practice staff, who were able to read free-text entries and apply their clinical judgement when completing data fields. The referral type is that recorded by the practice; of these, Urgent Suspected Cancer (also known as ‘Two Week Wait‘) referrals are an elective, although expedited, referral where a cancer is suspected; emergency referrals are a combination of emergency referrals directly from primary to secondary care and patient self-referrals to an emergency department. The characteristics of audited patients and participating practices in the NCDA are highly representative of the national incident cohort in respect of age, gender, and cancer diagnoses, and of all English practices.

### NEN classification

The cohort of NENs was identified following the SEER rare cancers list in ICD O3 [14] (Supplementary Table S1), based on the earlier RARECARE list [15]. This list was reviewed by NDRS experts to reflect recent changes in our understanding of NENs, for example, that Goblet Cell Carcinomas are no longer considered NENs. See Appendix 1 and Supplementary Fig. S1 for full details. Due to the selection of malignant-only tumours for the NCDA, we here include only NENs considered malignant under the ICD-10 system (henceforth ‘malignant NENs’). This led to the exclusion of most NENs of the appendix. The NCDA also did not select non-melanoma skin cancer, leading to the exclusion of all Merkel Cell Carcinomas of skin. Small cell carcinomas of the lung (SCLC) are also excluded, because although they represent NENs pathologically, they typically present with symptoms leading to management on the lung cancer diagnostic pathway. Tumours recorded as ‘death certificate only’, which made up less than 0.1% of NEN tumours in this dataset, were excluded [16]. It was our intent to exclude tumours in patients under 18 but none were present in the cohort. We also excluded tumours identified by either the practice or by data independently available to the NDRS as being referred via a screening route (1.1% of tumours).

### Analysis

The analysis largely follows that in prior NCDA work [10, 13]. The following key variables were used in the study, gender at diagnosis, age group (18–49, 50–64, 65–74, 75–84, and ≥85 years); ethnicity, grouped as White, non-White and not known due to the limited cohort size; socio-economic deprivation quintile (based on the 2019 Index of Multiple Deprivation (IMD) of the patient’s lower super output area of residence). Patients were classified into one of the following 11 primary NEN site groups: bladder, cancer of unknown primary, colon, lung, oesophageal, pancreatic, rectal, small intestine, stomach, thyroid, and other. Details of the ‘other’ cancer group are listed in Supplementary Table S2.

Tables describing the baseline characteristics of the study cohort were produced. A comparison of the distribution of the cancer sites in the NCDA and the national cancer registration NEN cohorts was made. The frequencies and proportions of key measures were summarised.

Primary care-led investigations were grouped into blood, urinary, imaging, endoscopy, and other tests. The number of pre-referral consultations and the number of comorbidities were categorised as 0, 1, 2, and ≥3. Four timescales were considered, namely patient interval (PI), primary care interval (PCI), diagnostic interval (DI), and referral to date informed whether the patient had cancer [17]. Interval times of <0 and >730 days were excluded, to exclude improbable long intervals, consistent with previous literature [18]; the numbers excluded from the analysis for this reason were: 12 (1.3%) for PI, 17 (1.8%) for PCI, 30 (3.3%) for DI and 12 (1.3%) for referral to date informed. Alarm symptoms were guided by the NICE 2015 clinical guidelines [19] and defined as: breast lump, post-menopausal bleeding, haematuria, haemoptysis, rectal bleeding, change in bowel habit (including diarrhoea and constipation), dysphagia, jaundice, weight loss, or lesions suspicious of melanoma [20].

Statistical tests were carried out on key metrics using chi-squared tests on categorical data and Mann–Whitney *U*-tests or Kruskal–Wallis tests on continuous variables that are not normally distributed.

## Results

Data were collected on 919 patients aged 18 years and over, diagnosed in 2018 with malignant NENs and registered with practices participating in the National Cancer Diagnosis Audit. A detailed description of the characteristics of the 1878 NCDA practices and their representativeness has been previously reported [10].

Table 1 shows the composition of the 2018 NCDA malignant NEN cohort. Cancers of the lung were most common (220, 23.9%) followed by small intestine (145, 15.8%) and pancreas (109, 11.9%). 48.4% of the cohort was female compared to 51.6% male. The mean and median age of the cohort were 65.4 and 67, respectively (males: 66.1 and 68, and females: 64.6 and 67). The cohort had an over-representation of the most deprived quintile (23.5%), although a trend across the other quintiles was not observed.

**Table 1:** Cohort composition and referral type that led most directly to the cancer diagnosis (*n* = 919)

	Total *n* (%)	USC^a^ *n* (%)	Urgent^b^ *n* (%)	Direct access *n* (%)	MDC^c^ or RDC^d^ *n* (%)	Routine *n* (%)	Screening *n* (%)	Emergency^e^ *n* (%)	To private care *n* (%)	Other *n* (%)	Not known *n* (%)
Total	919 (100.0)	414 (45.0)	31 (3.4)	15 (1.6)	5 (0.5)	72 (7.8)	36 (3.9)	169 (18.4)	13 (1.4)	74 (8.1)	90 (9.8)
Gender											
Male	474 (51.6)	210 (44.3)	18 (3.8)	4 (0.8)	3 (0.6)	32 (6.8)	15 (3.2)	93 (19.6)	7 (1.5)	46 (9.7)	46 (9.7)
Female	445 (48.4)	204 (45.8)	13 (2.9)	11 (2.5)	2 (0.4)	40 (9.0)	21 (4.7)	76 (17.1)	6 (1.3)	28 (6.3)	44 (9.9)
Age group, years											
18–49	123 (13.4)	44 (35.8)	7 (5.7)	0 (0.0)	0 (0.0)	22 (17.9)	1 (0.8)	28 (22.8)	1 (0.8)	6 (4.9)	14 (11.4)
50–64	260 (28.3)	105 (40.4)	6 (2.3)	5 (1.9)	2 (0.8)	19 (7.3)	24 (9.2)	45 (17.3)	8 (3.1)	20 (7.7)	26 (10.0)
65–74	277 (30.1)	132 (47.7)	10 (3.6)	7 (2.5)	1 (0.4)	17 (6.1)	9 (3.2)	45 (16.2)	1 (0.4)	28 (10.1)	27 (9.7)
75–84	209 (22.7)	108 (51.7)	6 (2.9)	3 (1.4)	1 (0.5)	11 (5.3)	2 (1.0)	39 (18.7)	3 (1.4)	17 (8.1)	19 (9.1)
85+	50 (5.4)	25 (50.0)	2 (4.0)	0 (0.0)	1 (2.0)	3 (6.0)	0 (0.0)	12 (24.0)	0 (0.0)	3 (6.0)	4 (8.0)
Ethnicity											
White	776 (84.4)	355 (45.7)	27 (3.5)	13 (1.7)	3 (0.4)	55 (7.1)	22 (2.8)	147 (18.9)	11 (1.4)	71 (9.1)	72 (9.3)
Non-white	106 (11.5)	47 (44.3)	4 (3.8)	1 (0.9)	2 (1.9)	12 (11.3)	6 (5.7)	16 (15.1)	1 (0.9)	3 (2.8)	14 (13.2)
Not known	37 (4.0)	12 (32.4)	0 (0.0)	1 (2.7)	0 (0.0)	5 (13.5)	8 (21.6)	6 (16.2)	1 (2.7)	0 (0.0)	4 (10.8)
IMD quintile											
1—most deprived	216 (23.5)	89 (41.2)	10 (4.6)	3 (1.4)	0 (0.0)	19 (8.8)	10 (4.6)	41 (19.0)	2 (0.9)	14 (6.5)	28 (13.0)
2	189 (20.6)	87 (46.0)	6 (3.2)	5 (2.6)	1 (0.5)	13 (6.9)	3 (1.6)	38 (20.1)	2 (1.1)	15 (7.9)	19 (10.1)
3	170 (18.5)	75 (44.1)	4 (2.4)	4 (2.4)	1 (0.6)	10 (5.9)	12 (7.1)	35 (20.6)	2 (1.2)	16 (9.4)	11 (6.5)
4	167 (18.2)	77 (46.1)	5 (3.0)	1 (0.6)	2 (1.2)	16 (9.6)	6 (3.6)	28 (16.8)	3 (1.8)	14 (8.4)	15 (9.0)
5—least deprived	177 (19.3)	86 (48.6)	6 (3.4)	2 (1.1)	1 (0.6)	14 (7.9)	5 (2.8)	27 (15.3)	4 (2.3)	15 (8.5)	17 (9.6)
Cancer site											
Bladder	27 (2.9)	17 (63.0)	0 (0.0)	0 (0.0)	0 (0.0)	1 (3.7)	0 (0.0)	4 (14.8)	0 (0.0)	3 (11.1)	2 (7.4)
Cancer of unknown primary	82 (8.9)	39 (47.6)	2 (2.4)	0 (0.0)	2 (2.4)	5 (6.1)	0 (0.0)	17 (20.7)	1 (1.2)	3 (3.7)	13 (15.9)
Colon	83 (9.0)	29 (34.9)	3 (3.6)	2 (2.4)	0 (0.0)	4 (4.8)	4 (4.8)	25 (30.1)	2 (2.4)	6 (7.2)	8 (9.6)
Lung	220 (23.9)	100 (45.5)	9 (4.1)	5 (2.3)	1 (0.5)	14 (6.4)	2 (0.9)	44 (20.0)	4 (1.8)	21 (9.5)	20 (9.1)
Oesophageal	32 (3.5)	23 (71.9)	1 (3.1)	3 (9.4)	0 (0.0)	1 (3.1)	0 (0.0)	2 (6.2)	0 (0.0)	0 (0.0)	2 (6.2)
Pancreatic	109 (11.9)	33 (30.3)	3 (2.8)	3 (2.8)	1 (0.9)	6 (5.5)	3 (2.8)	27 (24.8)	2 (1.8)	19 (17.4)	12 (11.0)
Rectal	65 (7.1)	27 (41.5)	1 (1.5)	0 (0.0)	0 (0.0)	8 (12.3)	21 (32.3)	3 (4.6)	1 (1.5)	3 (4.6)	1 (1.5)
Small intestine	145 (15.8)	74 (51.0)	6 (4.1)	0 (0.0)	1 (0.7)	12 (8.3)	4 (2.8)	28 (19.3)	2 (1.4)	9 (6.2)	9 (6.2)
Stomach	47 (5.1)	19 (40.4)	3 (6.4)	2 (4.3)	0 (0.0)	6 (12.8)	0 (0.0)	4 (8.5)	0 (0.0)	4 (8.5)	9 (19.1)
Thyroid	25 (2.7)	12 (48.0)	0 (0.0)	0 (0.0)	0 (0.0)	7 (28.0)	0 (0.0)	1 (4.0)	0 (0.0)	2 (8.0)	3 (12.0)
Other	84 (9.1)	41 (48.8)	3 (3.6)	0 (0.0)	0 (0.0)	8 (9.5)	2 (2.4)	14 (16.7)	1 (1.2)	4 (4.8)	11 (13.1)

^a^USC: Urgent Suspected Cancer.

^b^Urgent referral not for suspected cancer.

^c^MDC: Multi-disciplinary Diagnostic Centre.

^d^RDC: Rapid Diagnostic Centre.

^e^Emergency referral includes instances of patient self-referral.

The most frequently reported referral type was the Urgent Suspected Cancer (Two Week Wait) pathway (45.0%). 18.4% of the patients were referred as an emergency. A larger proportion of emergency presentations were observed in the youngest and oldest groups (age 18–49: 22.8% and 85 + : 24.0%). The least socio-economically deprived patients showed a higher proportion of urgent suspected cancer referrals (*p*-value = 0.664) and a lower proportion of emergency referrals (*p*-value = 0.659); however, these differences were not statistically significant.

Union for International Cancer Control (UICC) stage data were available for 614 patients (66.8%), of whom 171 (27.9%) were stage 1 and 242 (39.4%) were stage 4 (Supplementary Table S3). Over 90% of patients were native English speakers, 7.5% were housebound, and 7.2% were reported as having some form of communication difficulty.

The proportion of cases by cancer site was comparable to that for all malignant NENs, excluding skin, diagnosed in England in 2018, comparing malignant NENs and excluding screen-detected cases (Supplementary Table S4).

### Intervals and avoidable delays

The median PCI was 7 days, and the median DI was 54 days. 12.9% of patients had a PCI > 60 days, and 8.8% >90 days. There were greater proportions of females than males at both time points (15.9% vs. 10.0% and 12.0% vs. 5.9%, respectively). 31.1% of patients had a DI > 90 days, the greater proportion of whom were female (36.2% female vs. 26.3% male), Table 2 and Supplementary Fig. S2. Patients in the youngest age group, the least deprived, and those referred routinely had longer DIs (median 84 days, 62 days, and 158.5 days, respectively). Both the PCI and DI were highly heterogeneous by cancer type, with median PCI of 1.0 to 14.5 days (CUP and stomach, respectively) and median DI of 25.0 to 109.0 (oesophageal and thyroid, respectively). The least socio-economically deprived patients showed slightly longer PCI (*p*-value = 0.312) and DI (*p*-value = 0.245); however, these differences were not statistically significant.

**Table 2:** The distribution of the primary care interval (*n *= 565) and the diagnostic interval (*n *= 736) is described by patient characteristics, cancer site, and investigation status.

	Primary care interval^a^						Diagnostic interval^b^					
	*n*	25th centile	median	75th centile	% >60 days	% >90 days	*n*	25th centile	median	75th centile	% >60 days	% >90 days
Total	565	0	7.0	29.0	12.9	8.8	736	25.0	54.0	113.0	45.4	31.1
Gender												
Male	289	0	6.0	25.0	10.0	5.9	377	21.0	51.0	95.0	42.7	26.3
Female	276	0	8.0	37.0	15.9	12.0	359	26.5	57.0	121.0	48.2	36.2
Age group, years												
18–49	75	1.0	12.0	39.5	13.3	10.7	97	34.0	84.0	148.0	56.7	44.3
50–64	151	0	6.0	28.0	13.9	11.9	198	26.0	51.0	123.2	41.9	32.3
65–74	166	0	7.5	29.8	15.1	9.6	221	23.0	55.0	102.0	46.2	28.5
75–84	141	0	3.0	21.0	9.9	4.3	176	23.0	52.5	98.5	44.3	28.4
85+	32	0	3.5	14.0	9.4	6.2	44	15.5	39.0	81.5	36.4	20.5
Ethnicity												
White	481	0	7.0	28.0	12.1	7.9	633	25.0	53.0	113.0	45.2	30.8
Non-white	66	0	7.5	46.0	18.2	15.2	80	26.0	54.5	109.8	46.2	32.5
Not known	18	0	14.0	36.5	16.7	11.1	23	16.5	57.0	131.5	47.8	34.8
IMD												
1—most deprived	124	0	7.0	28.2	11.3	8.1	168	23.0	55.0	100.5	44.0	27.4
2	113	0	5.0	24.0	15.0	12.4	156	20.8	50.5	127.5	44.9	37.2
3	101	0	7.0	25.0	11.9	7.9	136	25.0	43.5	82.5	36.0	22.1
4	112	0	4.0	28.0	9.8	6.2	131	25.0	62.0	116.5	50.4	32.8
5—least deprived	115	0	11.0	40.0	16.5	9.6	145	30.0	62.0	116.0	51.7	35.9
Cancer site												
Bladder	18	0	7.0	19.8	11.1	5.6	22	33.0	44.5	60.5	27.3	18.2
Cancer of unknown primary	59	0	1.0	15.5	10.2	10.2	74	25.2	48.0	105.5	40.5	27.0
Colon	45	0	3.0	20.0	6.7	6.7	68	15.5	37.0	63.5	26.5	20.6
Lung	133	0	8.0	38.0	17.3	12.0	177	33.0	65.0	121.0	54.8	32.2
Oesophageal	26	0	5.5	17.8	3.8	0	29	14.0	25.0	48.0	24.1	3.4
Pancreatic	56	0	11.0	44.0	17.9	12.5	82	29.2	55.5	114.0	42.7	31.7
Rectal	32	0	2.0	23.0	9.4	9.4	38	26.2	42.0	94.8	39.5	26.3
Small intestine	95	0	4.0	22.5	13.7	8.4	121	21.0	76.0	142.0	57.0	47.1
Stomach	30	1.5	14.5	39.2	13.3	6.7	34	27.0	40.5	79.8	35.3	23.5
Thyroid	19	0	4.0	28.0	5.3	0	21	56.0	109.0	241.0	71.4	57.1
Other	52	0	13.5	38.5	13.5	7.7	70	25.2	49.5	98.0	42.9	28.6
Referral type												
USC^c^	374	0	7.0	31.0	13.6	8.8	384	29.0	56.0	103.0	45.6	28.1
Urgent^d^	25	0	3.0	21.0	8.0	4.0	26	70.0	135.0	252.0	80.8	61.5
Direct access test and upgrade	10	2.0	14.5	19.2	20.0	20.0	14	33.5	52.5	105.8	35.7	35.7
MDC^e^ or RDC^f^	3	7.5	15.0	21.0	0	0	3	105.5	149.0	180.0	100.0	66.7
Routine	59	0	13.0	39.0	20.3	15.3	64	99.5	158.5	276.0	87.5	79.7
Emergency^g^	74	0	1.0	7.0	5.4	4.1	155	8.0	27.0	61.5	26.5	16.1
To private care	11	0	5.0	17.0	9.1	9.1	12	33.0	81.0	140.0	66.7	33.3
Other	9	0	10.0	15.0	11.1	11.1	40	21.0	39.5	89.8	32.5	25.0
Not known	0	n/a	n/a	n/a	n/a	n/a	33	11.0	44.0	86.0	36.4	24.2
Primary care-led investigation												
Yes	407	1.0	13.0	39.0	16.7	11.1	435	37.0	66.0	124.5	54.7	38.4
No	151	0	0	3.0	3.3	3.3	294	13.0	30.5	75.8	32.0	20.7
Not known	7	0	2.0	9.0	0	0	7	40.0	45.0	60.5	28.6	14.3

^a^The PCI is defined as the number of days from the first presentation with symptoms deemed to be relevant to the subsequent diagnosis of cancer to the date of first referral from primary care for suspected cancer.

^b^The DI is defined as the number of days from the first relevant presentation to the date of diagnosis, as registered by the NDRS.

The PCI is calculated using the date of presentation and date of referral as supplied by GPs in the NCDA. The DI is calculated using the date of referral from the NCDA and the date of diagnosis from the NDRS. There are missing presentation and referral dates in the NCDA, but as all dates of diagnosis are complete, there are more patients with a valid DI than PCI.

Intervals are restricted to 0-730 days. Patients with a cancer diagnosis through screening are excluded.

^c^USC: Urgent Suspected Cancer.

^d^Urgent referral not for suspected cancer.

^e^MDC: Multi-disciplinary Diagnostic Centre.

^f^RDC: Rapid Diagnostic Centre.

^g^Emergency referral includes instances of patient self-referral.

Participating GPs perceived there to be one or more avoidable delays in diagnosis in 234 (25.5%) of cases. These were evenly distributed across the pre-presentation, primary care, and post-referral phases of the diagnostic pathway (Table 3). Avoidable delays were more common in the youngest (18–49) patient group, and roughly even between males and females. The patient interval and referral to date informed interval are shown in Supplementary Table S5.

**Table 3:** Avoidable delays

	Before presentation		Between presentation and referral		After referral		Delay in one or more time frames	
	Yes *n* (%)	Not known *n*	Yes *n* (%)	Not known *n*	Yes *n* (%)	Not known *n*	Yes *n* (%)	No/Not known *n*
Total	93 (12.4)	169	95 (12.0)	129	106 (14.0)	163	234 (25.5)	685
Gender								
Male	49 (12.7)	89	46 (11.4)	71	52 (13.5)	90	121 (25.5)	353
Female	44 (12.1)	80	49 (12.7)	58	54 (14.5)	73	113 (25.4)	332
Age group, years								
18–49	16 (16.5)	26	19 (18.3)	19	23 (22.3)	20	37 (30.1)	86
50–64	23 (11.2)	54	21 (9.6)	42	28 (13.5)	52	60 (23.1)	200
65–74	30 (12.9)	45	28 (11.6)	36	27 (11.7)	47	70 (25.3)	207
75–84	20 (11.6)	36	23 (12.7)	28	22 (13.0)	40	59 (28.2)	150
85+	4 (9.5)	8	4 (8.7)	4	6 (13.0)	4	8 (16.0)	42
Ethnicity								
White	80 (12.6)	140	81 (12.0)	100	90 (14.0)	131	202 (26.0)	574
Non-white	5 (5.7)	19	7 (8.1)	20	11 (12.8)	20	19 (17.9)	87
Not known	6 (27.3)	4	7 (30.4)	3	5 (23.8)	5	11 (42.3)	15
IMD quintile								
1—most deprived	24 (13.9)	43	24 (13.8)	42	19 (11.1)	45	55 (25.5)	161
2	23 (14.7)	33	20 (11.8)	20	27 (16.7)	27	56 (29.6)	133
3	13 (9.0)	25	14 (9.2)	17	21 (14.2)	22	38 (22.4)	132
4	14 (10.4)	33	18 (12.6)	24	17 (12.4)	30	36 (21.6)	131
5—least deprived	19 (13.4)	35	19 (12.6)	26	22 (15.9)	39	49 (27.7)	128
Cancer site								
Bladder	2 (8.7)	4	2 (7.7)	1	1 (4.3)	4	4 (14.8)	23
Cancer of unknown primary	9 (13.8)	17	7 (10.1)	13	9 (13.6)	16	21 (25.6)	61
Colon	7 (10.0)	13	9 (12.2)	9	11 (15.3)	11	19 (22.9)	64
Lung	27 (14.8)	38	27 (14.0)	27	21 (11.7)	40	63 (28.6)	157
Oesophageal	2 (7.1)	4	4 (13.3)	2	1 (3.7)	5	5 (15.6)	27
Pancreatic	14 (16.3)	23	12 (13.3)	19	15 (16.9)	20	30 (27.5)	79
Rectal	7 (14.0)	15	4 (8.0)	15	2 (4.2)	17	11 (16.9)	54
Small intestine	10 (8.4)	26	15 (12.0)	20	22 (18.5)	26	38 (26.2)	107
Stomach	1 (2.7)	10	5 (12.2)	6	12 (28.6)	5	15 (31.9)	32
Thyroid	4 (17.4)	2	3 (13.0)	2	6 (28.6)	4	7 (28.0)	18
Other	10 (14.9)	17	7 (10.1)	15	6 (8.7)	15	21 (25.0)	63

If there was a perceived avoidable delay in the patient receiving their diagnosis, information was gathered about where in the pathway this occurred. We define delay as an unnecessary prolongation of the time to reach a diagnosis that has potentially adverse consequences on outcomes.

### Investigations and consultations

Table 4 shows that 52.8% of patients had at least one primary care-led investigation, the most frequently ordered being blood tests and imaging. The proportion of patients investigated was greater in females (55.1% vs. 50.8%) and was notably greater in the use of imaging (35.0% vs. 25.6%). Imaging was also more commonly used in the youngest age group (18–49: 37.8% compared to 30.1% overall). Patients undergoing investigation in primary care had a longer median PCI (13 days vs. 0 days) and DI (66 days vs. 30.5 days) than those not investigated (Table 2).

**Table 4:** Number of primary care-led investigations that were ordered by the GP as part of the diagnostic assessment prior to referral

	No investigations *n* (%)	Any investigation *n* (%)	Not known *n*	Blood tests *n* (%)	Urinary tests *n* (%)	Imaging *n* (%)	Endoscopy *n* (%)	Symptomatic FIT *n* (%)	Other *n* (%)
Total	415 (47.2)	465 (52.8)	39	329 (37.4)	9 (1.0)	265 (30.1)	28 (3.2)	2 (0.2)	35 (4.0)
Gender									
Male	225 (49.2)	232 (50.8)	17	172 (37.6)	5 (1.1)	117 (25.6)	9 (2.0)	1 (0.2)	14 (3.1)
Female	190 (44.9)	233 (55.1)	22	157 (37.1)	4 (0.9)	148 (35.0)	19 (4.5)	1 (0.2)	21 (5.0)
Age group, years									
18–49	51 (42.9)	68 (57.1)	4	46 (38.7)	1 (0.8)	45 (37.8)	5 (4.2)	0 (0.0)	6 (5.0)
50–64	113 (46.5)	130 (53.5)	17	87 (35.8)	0 (0.0)	78 (32.1)	10 (4.1)	0 (0.0)	9 (3.7)
65–74	124 (47.1)	139 (52.9)	14	102 (38.8)	3 (1.1)	77 (29.3)	8 (3.0)	2 (0.8)	8 (3.0)
75–84	98 (47.8)	107 (52.2)	4	79 (38.5)	4 (2.0)	54 (26.3)	5 (2.4)	0 (0.0)	10 (4.9)
85+	29 (58.0)	21 (42.0)	0	15 (30.0)	1 (2.0)	11 (22.0)	0 (0.0)	0 (0.0)	2 (4.0)
Ethnicity									
White	362 (48.1)	390 (51.9)	24	274 (36.4)	8 (1.1)	222 (29.5)	24 (3.2)	2 (0.3)	30 (4.0)
Non-white	39 (40.2)	58 (59.8)	9	40 (41.2)	1 (1.0)	32 (33.0)	2 (2.1)	0 (0.0)	4 (4.1)
Not known	14 (45.2)	17 (54.8)	6	15 (48.4)	0 (0.0)	11 (35.5)	2 (6.5)	0 (0.0)	1 (3.2)
IMD quintile									
1—most deprived	101 (49.8)	102 (50.2)	13	70 (34.5)	1 (0.5)	58 (28.6)	10 (4.9)	0 (0.0)	7 (3.4)
2	85 (45.9)	100 (54.1)	4	76 (41.1)	1 (0.5)	62 (33.5)	9 (4.9)	2 (1.1)	5 (2.7)
3	77 (47.8)	84 (52.2)	9	56 (34.8)	2 (1.2)	50 (31.1)	2 (1.2)	0 (0.0)	7 (4.3)
4	78 (48.1)	84 (51.9)	5	60 (37.0)	2 (1.2)	44 (27.2)	4 (2.5)	0 (0.0)	7 (4.3)
5—least deprived	74 (43.8)	95 (56.2)	8	67 (39.6)	3 (1.8)	51 (30.2)	3 (1.8)	0 (0.0)	9 (5.3)
Cancer site									
Bladder	12 (46.2)	14 (53.8)	1	7 (26.9)	4 (15.4)	2 (7.7)	0 (0.0)	0 (0.0)	3 (11.5)
Cancer of unknown primary	28 (36.8)	48 (63.2)	6	36 (47.4)	0 (0.0)	35 (46.1)	1 (1.3)	0 (0.0)	4 (5.3)
Colon	45 (54.9)	37 (45.1)	1	33 (40.2)	1 (1.2)	17 (20.7)	4 (4.9)	0 (0.0)	2 (2.4)
Lung	89 (41.0)	128 (59.0)	3	62 (28.6)	0 (0.0)	113 (52.1)	3 (1.4)	0 (0.0)	8 (3.7)
Oesophageal	15 (48.4)	16 (51.6)	1	12 (38.7)	0 (0.0)	3 (9.7)	4 (12.9)	0 (0.0)	0 (0.0)
Pancreatic	59 (56.2)	46 (43.8)	4	37 (35.2)	2 (1.9)	26 (24.8)	1 (1.0)	0 (0.0)	2 (1.9)
Rectal	28 (54.9)	23 (45.1)	14	19 (37.3)	0 (0.0)	3 (5.9)	3 (5.9)	0 (0.0)	2 (3.9)
Small intestine	64 (45.7)	76 (54.3)	5	61 (43.6)	0 (0.0)	29 (20.7)	8 (5.7)	2 (1.4)	7 (5.0)
Stomach	17 (37.8)	28 (62.2)	2	24 (53.3)	0 (0.0)	9 (20.0)	3 (6.7)	0 (0.0)	1 (2.2)
Thyroid	11 (44.0)	14 (56.0)	0	11 (44.0)	0 (0.0)	8 (32.0)	0 (0.0)	0 (0.0)	3 (12.0)
Other	47 (57.3)	35 (42.7)	2	27 (32.9)	2 (2.4)	20 (24.4)	1 (1.2)	0 (0.0)	3 (3.7)

Of 633 patients with at least one recorded GP consultation (68.9%), 277 (43.8%) consulted once only, while 161 (25.4%) had three or more consultations (Supplementary Table S6). There was a record of safety netting being used in 337 (of 775 reported: 43.5%) patients (Supplementary Table S7). There was no clear pattern of socio-economic deprivation.

### Other measures

Table 5 summarises the comparative data from the NCDA for malignant NENs (excluding skin) and all cancers combined, for key features of the diagnostic pathway. The proportion of malignant NEN patients reporting alarm symptoms was lower than that for all cancers (28.6% against 32.5%, *p* < 0.05). More detailed information is included in the supplementary appendices on the number of alarm symptoms (Supplementary Table S8) and imaging by gender and cancer site (Supplementary Table S9).

**Table 5:** 2018 Malignant NENs compared to all malignant cancers

Outcome	NEN 2018		All cancers within NCDA 2018	
	Median (days)	90th centile (days)	Median (days)	90th centile (days)
Primary care interval (PCI)	7	74	2	56
Diagnostic interval (DI)	54	219	36	162

	%		%	
3+ consultations	25.4		18.8	
Emergency referred	18.4		13.2	
Urgent suspected cancer (Two Week Wait) referral	45.0		54.8	
Alarm symptoms	28.6		32.5	
Avoidable delays	25.5		23.2	
Investigated by a blood test	37.4		37.6	
Investigated by imaging	30.1		19.8	
Diagnosed as stage 4	39.4		25.5	
Safety netting recorded in notes	43.5		40.0	

## Discussion

In a large cohort of 919 patients diagnosed with malignant NENs in 2018 and included in the English NCDA, we found that the median diagnostic interval was 54 days, and the primary care interval was 7 days, compared to 36 days and 2 days for all cancers combined in the same dataset (*p*-values < 0.001), and that more patients consulted 3 or more times before referral [10] (25.4% vs. 18.8% for all cancers combined, *p*-value < 0.001). Substantial heterogeneity was observed by individual cancer site.

A strength of the study is that the NCDA methodology avoids the selection bias associated with surveys that recruit participants via social media and/or patient organisations. The study also has several weaknesses. Data on the patient interval—the period between the onset of symptoms and the first consultation with a GP were available for only 54.2% of cases, and we concluded that these data were not sufficiently robust for inclusion in the main analysis.

We examined the diagnostic pathway for malignant NENs only: the NCDA cohort criteria exclude those that are non-malignant or of uncertain or unknown nature. As such, we only considered those NENs registered by the NDRS with an ICD-10 ‘C code’, and not those with a ‘D code’ prefix. As a result, very few appendix NENs were included. Because the NCDA methodology excluded non-melanoma skin cancers, Merkel Cell Carcinoma of the skin was excluded from our dataset. Otherwise, the distribution by site of cases in the study mirrored that for England as a whole. We did not attempt to infer the functional status of the NENs included in this study, although we did have access to presenting symptoms. We instead chose to focus on presentation with alarm symptoms as these are a trigger to use of the urgent suspected cancer referral pathway and should have a direct effect on time to diagnosis [19, 21].

### Comparison with other literature

It can be challenging to compare studies on NENs due to the varying definitions adopted by different research groups. Some sub-categories that are unambiguously NENs from a pathological perspective may be excluded as patients with these conditions follow standard ‘mainstream’ cancer pathways (most notably small cell lung carcinoma, SCLC, but there are other less common examples) and are therefore considered irrelevant with respect to examination of NEN-specific care. Opinion may also differ over different types of lung tumours that are not specifically SCLC, and other groups where our understanding of the tumour has changed recently, such as pituitary adenomas.

The case mix seen in the population in our study is highly representative of that for all England, and the median age is the same as that reported by White et al. at 67 years [4]. The mean age at diagnosis is considerably higher, at 65.4 years, than that reported in two recent patient surveys, both of which included patients with the full range of neuroendocrine neoplasms. The first was an online survey of 303 patients identified through a patient organisation and by clinical nurse specialists in Neuroendocrine Tumour (NET) Units, Basuroy et al. [5]. The second study of 1928 patients was a global online and paper survey of patients from the International Neuroendocrine Cancer Alliance, a network of 18 independent charitable organisations and patient groups for individuals with neuroendocrine tumours from 15 countries around the world (922 participants from North America and 763 from Europe). The mean time since diagnosis was 5.2 years, and the mean age at diagnosis was 56.8 years, Singh et al. [6]. Moreover, 48.4% of our cohort were female, in contrast to 67.7% in the Basuroy sample [5] and 64% in the Singh sample [6]. The differences may be a reflection of their methods of survey subject recruitment. It is also notable that we found younger patients and women experienced the longest delays in diagnosis.

Despite the challenge in drawing comparisons, there are, however, considerable differences to be found with generally longer pathways featuring more consultations in patient survey studies. Basuroy et al. reported a mean total diagnostic interval (from onset of symptoms to diagnosis) of 53.8 months, a mean PCI of 37 months, and a mean secondary care interval of 17 months [5]. Patients reported a mean of 11 GP consultations before referral [5]. Singh et al. reported a similar total diagnostic interval, with a mean of 11.8 health care visits to a mean of 6.2 health care practitioners before diagnosis [6].

Shen et al. reported in a cohort study that of 9319 patients aged 65 years and over identified through the SEER MediCare database, 91.3% visited their primary care physician in the year before diagnosis. The median number of visits was 7 [22], with a mean of 22 visits to any speciality compared to 17 in non-cancer controls. In contrast, we found that only 25.4% of the patients in our study consulted a GP three or more times, though this was a greater proportion than for all cancers combined (18.8%).

Furthermore, we found that compared to all cancers in the English NCDA, fewer were diagnosed through urgent suspected cancer referral (45.0% vs. 54.8%, *p*-value < 0.001) and more through an emergency presentation (18.4% vs. 13.4%, *p*-value < 0.001). Shen reported that 35.0% of his cohort attended the Emergency Department in the 12 months before diagnosis (median 1 attendance), while a Canadian case-series of gastrointestinal neuroendocrine tumour patients reported that around half attended the emergency department in the 3 months pre-diagnosis [23]. Basuroy et al. reported that 31.4% (66/210) were admitted as an emergency from A&E, with 58.1% (122/210) being diagnosed via GP referral to a hospital clinic [5]; the latter figure is comparable to that of our finding for all non-emergency hospital clinic referrals.

We confirmed that the majority of patients (44.7%) present with non-alarm symptoms, though this is only a little greater than the proportion for all cancers combined (41.0%). Possibly relatedly, avoidable delays were reported in 25.5% of cases, compared to 23.2% for all cancers combined. Nevertheless, an appreciable minority of patients presented with alarm symptoms, when the prevailing narrative is of an incidental finding or presentation with non-specific symptoms. These differences are likely explained by the recall and survivorship biases that can be associated with symptom self-report [24].

The use of diagnostic imaging was more extensive than for all cancers (30.1% vs. 19.8%, *p*-value < 0.001), and this difference was even more pronounced for women (35.0% vs. 19.7%, *p*-value < 0.001), although there was no clear pattern by socio-economic deprivation. Breaking down imaging investigations by gender, we found that two-thirds of the excess imaging investigations in women related to lung NENs. A recent analysis of lung NENs diagnosed between 1988 and 2015 and recorded in the SEER database reported a gender balance in overall incidence, but when SCLCs were excluded, as we did for our cohort, it was found that 56.1% of lung NENs occur in women [25]. The median PCI and DI were longer for those patients undergoing investigations than for those not investigated (13 days vs. 0 days), but these are consistent with the differences observed for all cancers in the 2014 and 2018 NCDA [10, 13].

### Implications for practice

While broadly in agreement with prior population-based studies, compared to previous survey-based studies, we found much shorter median primary care and diagnostic intervals for patients with NENs in England, and only a quarter of patients consulted their GP 3 or more times before referral. Nevertheless, these intervals are much longer than for all cancers combined, and the general picture in other metrics examined is of a more difficult diagnostic journey for NEN patients. This lends support to the recommendations of the Neuroendocrine Cancer UK consensus statement on the need for greater awareness among clinicians on the clinical presentation of NENs, use of Rapid Diagnostic Centres, and better access to diagnostic testing and specialist advice [9]. These last may be positively impacted by a recent policy announcement on elective care reform in England [26], which promotes investment in both.

Considering the median length of the primary care interval relative to the diagnostic interval, most of the time expended prior to diagnosis occurs after referral. By its design, our study could not examine the reasons for delays in NEN diagnosis after a primary care referral, but they likely reflect the need for specialist diagnostic expertise and testing facilities, and limitations in the availability of radiology and neuroendocrine expertise have been cited as contributory factors [9]. Given that often NEN symptoms are non-organ specific, the need for assessment by specialties beyond that to which the patient is initially referred is also likely, though this remains a hypothesis to be examined by future research.

### Future research

The data used for this study were collected in 2018, prior to the full implementation of non-specific symptom referral pathways [27], but also prior to COVID and the greater restrictiveness post-COVID of some investigative pathways, such as for gastrointestinal endoscopy [28, 29]. These changes in service provision could plausibly have impacted the timeliness of NEN diagnosis, for better or worse, and merit an updated examination of diagnostic pathways for NENs along the lines of the NCDA model in both primary and secondary care, to better understand where improvements are feasible.

## Conclusions

To our knowledge, this is the first attempt at comprehensively describing the diagnostic process of patients with neuroendocrine tumours from a population-based primary care perspective, using a large and representative cohort of patients diagnosed with NEN in 2018. We found longer PCI and DI than for all cancers combined, but substantially shorter than those previously reported by patient surveys, with considerably fewer GP consultations before referral. The greater proportion of the time between first presentation and diagnosis is expended in secondary care, highlighting the potential to improve patient outcomes by optimising diagnostic management post-referral. Overall, the findings do not seem to support targeting efforts to improve the diagnostic process for NENs, specifically; instead, a holistic approach that supports the diagnostic management of patients with new symptoms is justified to improve the diagnosis of both NENs and patients with other cancers.

## Supplementary information

Supplement.

## Data Availability

Data for this audit are based on patient-level information collected by the NHS, as part of the care and support of patients with cancer. The data are collated, maintained, and quality assured by the National Cancer Registration and Analysis Service, which is now part of NHS England (it was part of Public Health England during the data-collection phase of the audit). Data for the National Cancer Diagnosis Audit are held by NHS England and can be made available for approved users through the Data Access Request Service: https://digital.nhs.uk/services/data-access-request-service-dars.

## Code availability

All analysis was performed in R v4.5.0. The R code used for this analysis is available on the NDRS GitHub page and can also be obtained from the authors upon request.
