# Supplementary material for: Characterisation of adult patients with neuroendocrine neoplasms and their journey to diagnosis
Source: BJC Rep. 2026 Jan 13;4:1. doi: 10.1038/s44276-025-00198-3 (PMC12800212; doi:10.1038/s44276-025-00198-3)
Supplement: Supplementary file 1 — Supplement. [file 44276_2025_198_MOESM1_ESM.docx]

**Supplementary Material**

**Characterisation of adult patients with neuroendocrine neoplasms and their journey to diagnosis**

Busani Ndlela,^1^* Ruth Swann,^1,2^* Georgios Lyratzopoulos^3^, Sally Vernon^1^, Brian Rous^1^, Sean McPhail^1^,

Greg Rubin^4^

**Appendix 1.** Neuroendocrine Neoplasms (NENs), National Disease Registration Service definition

This definition considers certain neoplasms based on their ICD O3 morphology, behaviour, and tumour topography here as NENs. Other combinations may, perhaps, have been categorised as such elsewhere. Additionally, different analytical subsets within this pathological definition may be useful for different purposes. It is for example common to exclude small cell lung cancer from the analysis as these are typically managed on lung cancer clinical pathways.

There are about 10,000 tumours a year diagnosed in England in the broadest analytical definition of NENs.

Supplementary Figure S1 shows a broad classification of NENs, the broad subtypes and more detailed individual subtype. A small proportion of NENs, in this definition, are neither Neuroendocrine Carcinomas (NECs) nor Neuroendocrine Tumours (NETs) – these are medullary carcinomas and paraganglioma/phaeochromocytomas.

**Supplementary** **Figure S1**. Neuroendocrine Neoplasms (NENs) hierarchy

| NENs | | | |
| --- | --- | --- | --- |
| Epithelial NENs | | | Neural NENs |
| NEC | NET |  |  |
| Small cell, large cell, merkel cell | Carcinoids, NETs, pituitary adenomas, pancreatic 'omas | Medullary carcinoma | Paraganglioma / phaeochromocytoma |

**Supplementary Table S1**. ICD O3 morphology and behaviour codes for the superset of groups considered for categorisation as NENs.

| **Morphology** | **Behaviour** | **Description** | **Broad Subtype** | **Cohort** |
| --- | --- | --- | --- | --- |
| 8002 | 3 | Malignant tumour, small cell type | NEC | Small cell tumour |
| 8013 | 3 | Large cell neuroendocrine carcinoma | NEC | Large cell neuroendocrine carcinoma |
| 8041 | 3 | Small cell carcinoma, NOS | NEC | Small cell (lung) |
| 8042 | 3 | Oat cell carcinoma | NEC | Small cell (lung) |
| 8043 | 3 | Small cell carcinoma, fusiform cell | NEC | Small cell (lung) |
| 8044 | 3 | Small cell carcinoma, intermediate cell | NEC | Hypercalcaemic reassignment |
| 8045 | 3 | Combined small cell carcinoma | NEC | Small cell (lung) |
| 8150 | 0 | Pancreatic neuroendocrine microadenoma | NET | NET |
| 8150 | 3 | Pancreatic neuroendocrine tumour, nonfunctioning | NET | NET |
| 8151 | 3 | Insulinoma, NOS | NET | NET |
| 8152 | 3 | Glucagonoma | NET | NET |
| 8153 | 3 | Gastrinoma | NET | NET |
| 8154 | 3 | Mixed neuroendocrine non-neuroendocrine neoplasm (MiNEN) | NET | NET |
| 8155 | 3 | Vipoma | NET | NET |
| 8156 | 3 | Somatostatinoma | NET | NET |
| 8157 | 3 | Enteroglucagonoma | NET | Enteroglucagonoma |
| 8158 | 3 | ACTH-producing tumour | NET | ACTH-producing tumour |
| 8240 | 3 | Neuroendocrine tumour, NOS | NET | NET |
| 8241 | 3 | Enterochromaffin cell carcinoid | NET | NET |
| 8242 | 3 | Enterochromaffin-like cell tumour | NET | NET |
| 8243 | 3 | Goblet cell carcinoid | NET | Goblet cell carcinoid |
| 8244 | 3 | Mixed adenoneuroendocrine carcinoma | NET | NET |
| 8245 | 1 | Tubular carcinoid | NET | NET |
| 8245 | 3 | Adenocarcinoid tumour | NET | NET |
| 8246 | 3 | Neuroendocrine carcinoma, NOS | NEC | NEC NOS |
| 8247 | 3 | Merkel cell carcinoma | NEC | Merkel cell carcinoma |
| 8248 | 1 | Apudoma | NET | Apudoma |
| 8249 | 3 | Neuroendocrine tumour, grade 2 | NET | NET |
| 8270 | 0 | Chromophobe adenoma | NET | PitNET |
| 8270 | 1 | Chromophobe adenoma | NET | PitNET |
| 8270 | 3 | Chromophobe adenoma | NET | PitNET |
| 8271 | 0 | Prolactinoma | NET | PitNET |
| 8271 | 1 | Prolactinoma | NET | PitNET |
| 8271 | 3 | Prolactinoma | NET | PitNET |
| 8272 | 0 | Pituitary adenoma | NET | PitNET |
| 8272 | 1 | Pituitary adenoma | NET | PitNET |
| 8272 | 3 | Pituitary adenoma | NET | PitNET |
| 8280 | 0 | Acidophil adenoma | NET | PitNET |
| 8280 | 1 | Acidophil adenoma | NET | PitNET |
| 8280 | 3 | Acidophil adenoma | NET | PitNET |
| 8281 | 0 | Mixed acidophil-basophil adenoma | NET | PitNET |
| 8281 | 1 | Mixed acidophil-basophil adenoma | NET | PitNET |
| 8281 | 3 | Mixed acidophil-basophil adenoma | NET | PitNET |
| 8345 | 3 | Medullary carcinoma with amyloid stroma | Other | Medullary carcinoma |
| 8346 | 3 | Mixed medullary-follicular carcinoma | Other | Medullary carcinoma |
| 8347 | 3 | Mixed medullary-papillary carcinoma | Other | Medullary carcinoma |
| 8510 | 3 | Medullary carcinoma NOS | Other | Medullary carcinoma |
| 8512 | 3 | Medullary carcinoma with lymphoid stroma | Other | Medullary carcinoma |
| 8574 | 3 | Adenocarcinoma with neuroendocrine differentiation | NET | Adenocarcinoma with neuroendocrine differentiation |
| 8680 | 0 | Paraganglioma | Other | Paraganglioma |
| 8680 | 1 | Paraganglioma | Other | Paraganglioma |
| 8680 | 3 | Paraganglioma | Other | Paraganglioma |
| 8693 | 0 | Extra-adrenal paraganglioma | Other | Paraganglioma |
| 8693 | 1 | Extra-adrenal paraganglioma | Other | Paraganglioma |
| 8693 | 3 | Extra-adrenal paraganglioma | Other | Paraganglioma |
| 8700 | 0 | Pheochromocytoma | Other | Paraganglioma |
| 8700 | 1 | Pheochromocytoma | Other | Paraganglioma |
| 8700 | 3 | Pheochromocytoma | Other | Paraganglioma |
| 9091 | 1 | Strumal carcinoid | NET | Strumal carcinoid |

Supplementary Table S1 shows the ICD O3 morphology codes with the potential to be included as NENS. All tumours with these combinations of morphology and behaviour are included in our NENs cohort, which the exception of tumours matching the following conditions, which are excluded: cohort = ‘Hypercalcaemic reassignment’; cohort = ‘Goblet cell carcinoid’; cohort = ‘Medullary carcinoma’ & topography not equal to ICD O3 C739 (thyroid); and finally cohort = ‘Adenocarcinoma with neuroendocrine differentiation’.

Additionally, while we do count small cell lung tumours (SCLC) as being pathologically NENs we exclude them from the analysis in this study as the patients tend to follow lung referral pathways rather than those pathways being characteristic of NENs. That is, all tumours with ICD-10 topography of C33 or C34 and cohort = ‘Small cell (lung)’ are excluded.

**Supplementary Table S2**. Cancer sites included in the ‘other’ cancer group

| **Cancer** | **Count** |
| --- | --- |
| Adrenal gland | 7 |
| Anus | 3 |
| Brain | 3 |
| Breast | 5 |
| Cervix uteri | 6 |
| Head and neck - hypopharynx | 1 |
| Head and neck - larynx | 4 |
| Head and neck - major salivary glands | 3 |
| Head and neck - nasal cavity and sinus | 2 |
| Head and neck - nasopharynx | 1 |
| Head and neck - oropharynx | 1 |
| Heart, mediastinum, pleura, other and ill-defined | 1 |
| Kidney | 2 |
| Liver and biliary tract | 9 |
| Other and ill-defined digestive organs | 6 |
| Ovarian | 8 |
| Prostate | 8 |
| Renal pelvis | 1 |
| Soft tissue | 5 |
| Thymus | 3 |
| Uterus | 3 |
| Vagina | 2 |

**Supplementary Table S3.** Patient characteristics

| **Variable** | **n (%)** |
| --- | --- |
| **Union for International Cancer Control (UICC) cancer stage group** | |
| 1 | 171 (27.9) |
| 2 | 85 (13.8) |
| 3 | 116 (18.9) |
| 4 | 242 (39.4) |
| Not known | 305 |
| **Ethnicity** |  |
| White | 776 (88.0) |
| Asian | 43 (4.9) |
| Black | 43 (4.9) |
| Mixed | 5 (0.6) |
| Other | 15 (1.7) |
| Not known | 37 |
| **IMD quintile** |  |
| 1 - most deprived | 216 (23.5) |
| 2 | 189 (20.6) |
| 3 | 170 (18.5) |
| 4 | 167 (18.2) |
| 5 - least deprived | 177 (19.3) |
| **Language** |  |
| Is a native English speaker | 795 (92.8) |
| English is not the patient’s mother tongue but they are very fluent in English | 44 (5.1) |
| English not mother tongue and communication only possible through translator | 10 (1.2) |
| English not mother tongue and patient not fluent in English | 8 (0.9) |
| Not known | 62 |
| **Communication difficulty** |  |
| No difficulty | 721 (92.8) |
| Cognitive impairment | 13 (1.7) |
| Language barrier | 11 (1.4) |
| Hearing impairment | 17 (2.2) |
| Vision impairment | 8 (1.0) |
| Severe longstanding mental illness | 3 (0.4) |
| Learning difficulty | 3 (0.4) |
| Speech impairment | 5 (0.6) |
| Other | 1 (0.1) |
| Not known | 142 |
| **Housebound status** |  |
| The patient is not considered housebound | 773 (92.5) |
| The patient is considered housebound | 63 (7.5) |
| Not known | 83 |
| **Living arrangements** |  |
| Co-habiting | 514 (76.8) |
| Living alone | 145 (21.7) |
| In residential or nursing home | 10 (1.5) |
| **Number of comorbidities** |  |
| 0 | 186 (21.1) |
| 1 | 278 (31.5) |
| 2 | 201 (22.8) |
| 3+ | 217 (24.6) |
| Not known | 37 |
| **Type of comorbidity** |  |
| No comorbidity | 186 (21.1) |
| Hypertension | 327 (37.1) |
| Cardiovascular disease | 181 (20.5) |
| Arthritis / musculoskeletal disease | 162 (18.4) |
| Diabetes | 177 (20.1) |
| COPD or other chronic respiratory illness | 145 (16.4) |
| Previous cancer | 129 (14.6) |
| Cerebrovascular disease | 48 (5.4) |
| Cognitive impairment | 20 (2.3) |
| Severe longstanding mental illness | 31 (3.5) |
| Longstanding physical disability | 11 (1.2) |
| Other comorbidity | 202 (22.9) |
| Not known | 37 |

**Supplementary Table S4.** Proportion and number of cases by cancer site for England malignant and non-malignant NENs, England malignant NENs with screening, England malignant without screening and NCDA malignant NENs diagnosed in 2018

|  | **England malignant and non-malignant NENs**  **n (%)** | **England malignant NENs with screening**  **n (%)** | **England malignant NENs without screening**  **n (%)** | **NCDA malignant NENs**  **n (%)** |
| --- | --- | --- | --- | --- |
| **Gender** |  |  |  |  |
| Male | 2955 (51.0) | 2352 (52.3) | 2335 (52.3) | 474 (51.6) |
| Female | 2844 (49.0) | 2147 (47.7) | 2126 (47.7) | 445 (48.4) |
| **Age group, years** |  |  |  |  |
| 18-49 | 1116 (19.2) | 577 (12.8) | 574 (12.9) | 123 (13.4) |
| 50-64 | 1617 (27.9) | 1256 (27.9) | 1239 (27.8) | 260 (28.3) |
| 65-74 | 1634 (28.2) | 1419 (31.5) | 1403 (31.5) | 277 (30.1) |
| 75-84 | 1148 (19.8) | 1002 (22.3) | 1000 (22.4) | 209 (22.7) |
| 85+ | 284 (4.9) | 245 (5.4) | 245 (5.5) | 50 (5.4) |
| **Ethnicity** |  |  |  |  |
| White | 4927 (85.0) | 3910 (86.9) | 3878 (86.9) | 776 (84.4) |
| Non-white | 556 (9.6) | 387 (8.6) | 383 (8.6) | 106 (11.5) |
| Not known | 316 (5.4) | 202 (4.5) | 200 (4.5) | 37 (4.0) |
| **IMD quintile** |  |  |  |  |
| 1 - most deprived | 1094 (18.9) | 846 (18.8) | 842 (18.9) | 216 (23.5) |
| 2 | 1077 (18.6) | 840 (18.7) | 833 (18.7) | 189 (20.6) |
| 3 | 1244 (21.5) | 960 (21.3) | 947 (21.2) | 170 (18.5) |
| 4 | 1204 (20.8) | 939 (20.9) | 933 (20.9) | 167 (18.2) |
| 5 - least deprived | 1180 (20.3) | 914 (20.3) | 906 (20.3) | 177 (19.3) |
| **Cancer site** |  |  |  |  |
| Bladder | 181 (3.1) | 181 (4.0) | 181 (4.1) | 27 (2.9) |
| Cancer of unknown primary | 377 (6.5) | 377 (8.4) | 377 (8.5) | 82 (8.9) |
| Colon | 314 (5.4) | 314 (7.0) | 304 (6.8) | 83 (9.0) |
| Lung | 1081 (18.6) | 1081 (24.0) | 1081 (24.2) | 220 (23.9) |
| Oesophageal | 144 (2.5) | 144 (3.2) | 144 (3.2) | 32 (3.5) |
| Pancreatic | 552 (9.5) | 552 (12.3) | 552 (12.4) | 109 (11.9) |
| Rectal | 264 (4.6) | 264 (5.9) | 247 (5.5) | 65 (7.1) |
| Small intestine | 798 (13.8) | 798 (17.7) | 798 (17.9) | 145 (15.8) |
| Stomach | 243 (4.2) | 243 (5.4) | 243 (5.4) | 47 (5.1) |
| Thyroid | 102 (1.8) | 102 (2.3) | 102 (2.3) | 25 (2.7) |
| Other (malignant) | 443 (7.6) | 443 (9.8) | 432 (9.7) | 84 (9.1) |
| **Non-malignant cancer sites** | |  |  |  |
| Benign neoplasm of other and unspecified endocrine glands | 809 (14.0) | n/a | n/a | n/a |
| Neoplasm of uncertain or unknown behaviour of oral cavity and digestive organs | 435 (7.5) | n/a | n/a | n/a |
| Neoplasm of uncertain or unknown behaviour of endocrine glands | 33 (0.6) | n/a | n/a | n/a |
| Other non-malignant | 23 (0.4) | n/a | n/a | n/a |

**Supplementary** **Figure S2**. a) Primary care interval and b) diagnostic interval by age group, cancer site and referral type

a)
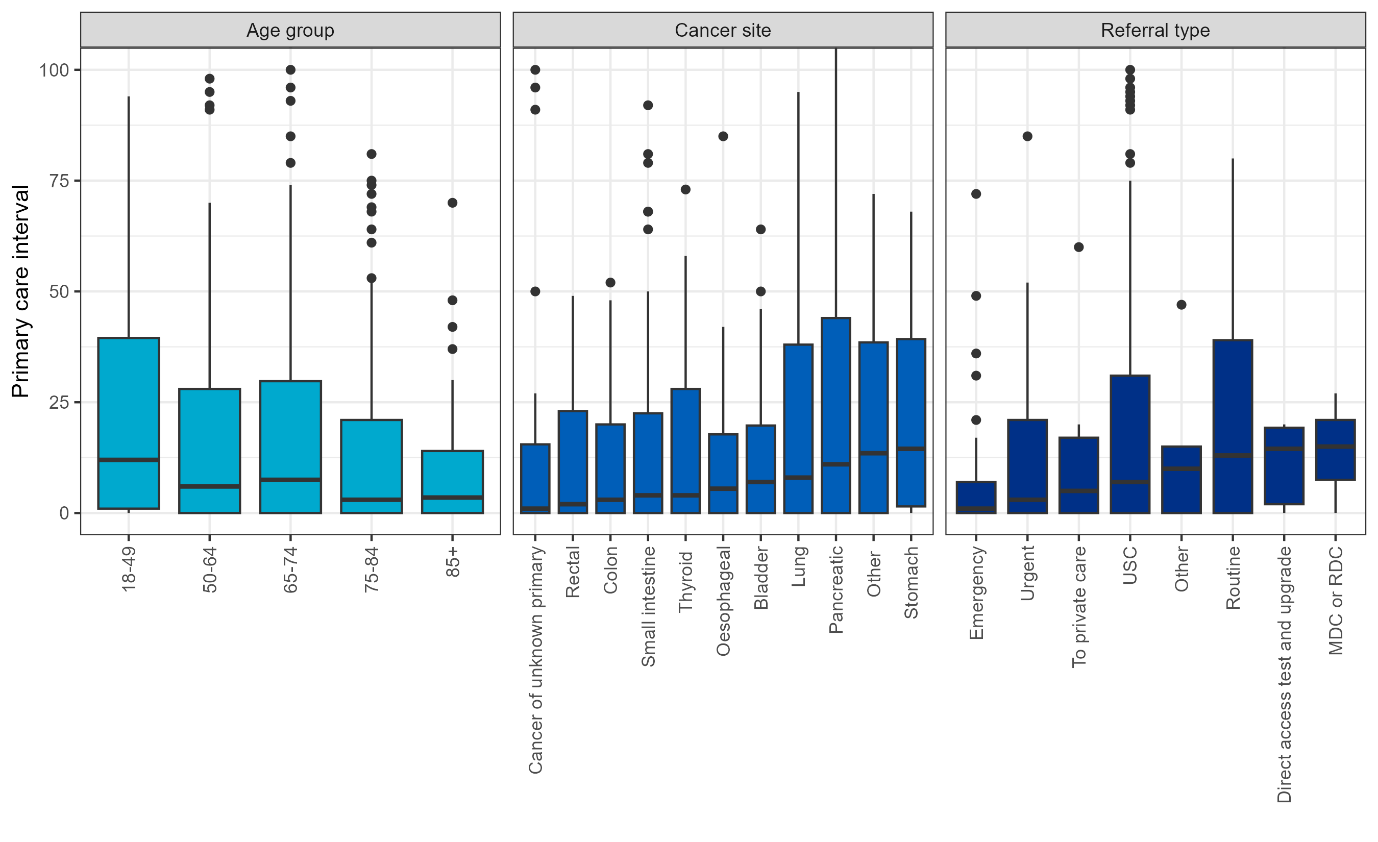


b)


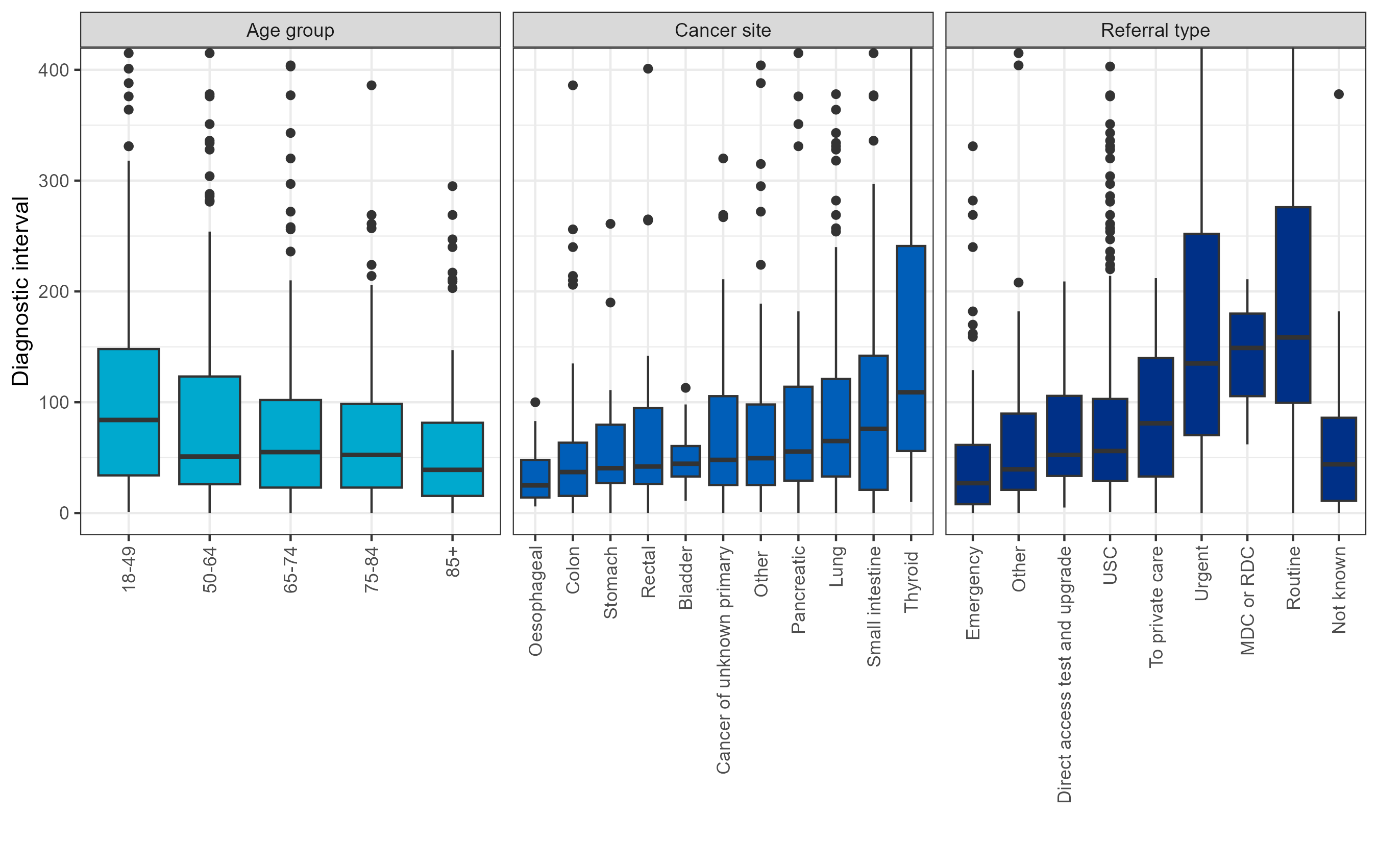


The PCI is defined as the number of days from first presentation with symptoms deemed to be relevant to the subsequent diagnosis of cancer to the date of first referral from primary care for suspected cancer.

The DI is defined as the number of days from first relevant presentation to the date of diagnosis, as registered by the NDRS.

Intervals are restricted to 0-730 days. Patients with a cancer diagnosed through screening are excluded.

USC: Urgent Suspected Cancer

Urgent referral not for suspected cancer

MDC: Multi-disciplinary Diagnostic Centre

RDC: Rapid Diagnostic Centre

Emergency referral includes instances of patient self-referral

**Supplementary Table S5.** The distribution of the patient interval (n = 498) and referral to date informed interval (n=327) by patient characteristic and cancer diagnosis groups.

|  | **Patient interval^a^** | | | | | | | | **Referral to date informed interval^b^** | | | | | |
| --- | --- | --- | --- | --- | --- | --- | --- | --- | --- | --- | --- | --- | --- | --- |
|  | **n** | **25th centile** | **median** | **75th centile** | **0-14 days**  **%** | **15+ days**  **%** | **%**  **>60 days** | **%**  **>90 days** | **n** | **25th centile** | **median** | **75th centile** | **% >60 days** | **% >90 days** |
| **Total** | 498 | 0 | 1.5 | 22.8 | 70.5 | 29.5 | 14.9 | 8.6 | 327 | 27.0 | 44.0 | 84.0 | 36.7 | 23.2 |
| **Gender** |  |  |  |  |  |  |  |  |  |  |  |  |  |  |
| Male | 258 | 0 | 2.0 | 24.8 | 68.6 | 31.4 | 12.8 | 7.8 | 169 | 28.0 | 48.0 | 90.0 | 39.1 | 24.9 |
| Female | 240 | 0 | 1.0 | 20.2 | 72.5 | 27.5 | 17.1 | 9.6 | 158 | 26.0 | 42.0 | 76.0 | 34.2 | 21.5 |
| **Age group, years** | |  |  |  |  |  |  |  |  |  |  |  |  |  |
| 18-49 | 64 | 0 | 0 | 20.2 | 71.9 | 28.1 | 15.6 | 7.8 | 37 | 25.0 | 34.0 | 90.0 | 29.7 | 24.3 |
| 50-64 | 136 | 0 | 2.0 | 28.0 | 69.1 | 30.9 | 17.6 | 10.3 | 89 | 27.0 | 41.0 | 96.0 | 42.7 | 27.0 |
| 65-74 | 151 | 0 | 4.0 | 25.5 | 68.9 | 31.1 | 14.6 | 10.6 | 101 | 26.0 | 44.0 | 73.0 | 32.7 | 21.8 |
| 75-84 | 116 | 0 | 0 | 22.2 | 73.3 | 26.7 | 12.9 | 6.0 | 78 | 31.2 | 54.5 | 75.0 | 42.3 | 23.1 |
| 85+ | 31 | 0 | 1.0 | 21.0 | 71.0 | 29.0 | 9.7 | 3.2 | 22 | 30.0 | 40.0 | 55.8 | 22.7 | 13.6 |
| **Ethnicity** |  |  |  |  |  |  |  |  |  |  |  |  |  |  |
| White | 427 | 0 | 2.0 | 24.0 | 69.6 | 30.4 | 14.8 | 8.0 | 282 | 26.2 | 43.0 | 86.0 | 37.2 | 23.8 |
| Non-white | 56 | 0 | 0 | 11.5 | 76.8 | 23.2 | 12.5 | 8.9 | 37 | 30.0 | 46.0 | 77.0 | 29.7 | 16.2 |
| Not known | 13 | 0 | 7.0 | 14.0 | 84.6 | 15.4 | 15.4 | 15.4 | 7 | 34.5 | 49.0 | 82.0 | 42.9 | 28.6 |
| **IMD quintile** |  |  |  |  |  |  |  |  |  |  |  |  |  |  |
| 1 - most deprived | 115 | 0 | 1.0 | 23.0 | 69.6 | 30.4 | 15.7 | 7.8 | 73 | 28.0 | 41.0 | 72.0 | 32.9 | 17.8 |
| 2 | 104 | 0 | 2.0 | 22.2 | 69.2 | 30.8 | 11.5 | 6.7 | 71 | 23.0 | 43.0 | 79.0 | 35.2 | 22.5 |
| 3 | 94 | 0 | 0 | 26.0 | 69.1 | 30.9 | 14.9 | 8.5 | 60 | 25.0 | 48.5 | 84.5 | 40.0 | 23.3 |
| 4 | 90 | 0 | 1.0 | 20.2 | 72.2 | 27.8 | 14.4 | 6.7 | 58 | 27.0 | 42.5 | 116.0 | 43.1 | 32.8 |
| 5 - least deprived | 95 | 0 | 2.0 | 21.0 | 72.6 | 27.4 | 17.9 | 13.7 | 65 | 30.0 | 45.0 | 82.0 | 33.8 | 21.5 |
| **Cancer site** |  |  |  |  |  |  |  |  |  |  |  |  |  |  |
| Bladder | 17 | 0 | 0 | 2.0 | 88.2 | 11.8 | 0 | 0 | 12 | 12.8 | 30.5 | 47.0 | 25.0 | 0 |
| Cancer of unknown primary | 52 | 0 | 0.5 | 28.8 | 65.4 | 34.6 | 7.7 | 1.9 | 30 | 30.0 | 42.5 | 72.5 | 36.7 | 20.0 |
| Colon | 43 | 0 | 2.0 | 13.5 | 81.4 | 18.6 | 11.6 | 7.0 | 24 | 35.2 | 54.5 | 87.2 | 41.7 | 25.0 |
| Lung | 122 | 0 | 2.0 | 27.8 | 67.2 | 32.8 | 14.8 | 8.2 | 76 | 19.8 | 34.0 | 59.0 | 25.0 | 17.1 |
| Oesophageal | 24 | 0 | 10.0 | 97.5 | 54.2 | 45.8 | 33.3 | 29.2 | 19 | 14.0 | 22.0 | 46.0 | 15.8 | 5.3 |
| Pancreatic | 53 | 0 | 2.0 | 11.0 | 77.4 | 22.6 | 9.4 | 3.8 | 26 | 31.5 | 46.0 | 83.5 | 46.2 | 23.1 |
| Rectal | 28 | 0 | 6.0 | 29.0 | 67.9 | 32.1 | 17.9 | 14.3 | 24 | 27.0 | 34.5 | 79.2 | 29.2 | 20.8 |
| Small intestine | 79 | 0 | 2.0 | 23.0 | 68.4 | 31.6 | 19 | 12.7 | 54 | 42.2 | 80.0 | 148.5 | 61.1 | 48.1 |
| Stomach | 22 | 0 | 0 | 10.8 | 77.3 | 22.7 | 13.6 | 4.5 | 16 | 25.8 | 47.5 | 76.2 | 37.5 | 25.0 |
| Thyroid | 10 | 0.2 | 17.5 | 67.0 | 50.0 | 50.0 | 30 | 10.0 | 12 | 44.0 | 76.0 | 132.2 | 58.3 | 33.3 |
| Other | 48 | 0 | 0 | 15.2 | 75.0 | 25.0 | 16.7 | 8.3 | 34 | 25.8 | 38.0 | 61.8 | 26.5 | 14.7 |
| **Referral type** |  |  |  |  |  |  |  |  |  |  |  |  |  |  |
| USC^c^ | 271 | 0 | 3.0 | 33.5 | 62.4 | 37.6 | 18.8 | 11.8 | 327 | 27.0 | 44.0 | 84.0 | 36.7 | 23.2 |
| Urgent^d^ | 19 | 5.5 | 30.0 | 72.5 | 36.8 | 63.2 | 36.8 | 15.8 | 0 | n/a | n/a | n/a | n/a | n/a |
| Direct access test and upgrade | 10 | 1.0 | 8.0 | 70.5 | 60.0 | 40.0 | 30.0 | 20.0 | 0 | n/a | n/a | n/a | n/a | n/a |
| MDC^e^ or RDC^f^ | 2 | 2.8 | 5.5 | 8.2 | 100.0 | 0 | 0 | 0 | 0 | n/a | n/a | n/a | n/a | n/a |
| Routine | 35 | 0 | 0 | 37.0 | 71.4 | 28.6 | 22.9 | 14.3 | 0 | n/a | n/a | n/a | n/a | n/a |
| Emergency^g^ | 117 | 0 | 0 | 5.0 | 90.6 | 9.4 | 0 | 0 | 0 | n/a | n/a | n/a | n/a | n/a |
| To private care | 8 | 5.5 | 12.0 | 23.0 | 62.5 | 37.5 | 12.5 | 0 | 0 | n/a | n/a | n/a | n/a | n/a |
| Other | 18 | 0 | 0 | 3.5 | 83.3 | 16.7 | 11.1 | 0 | 0 | n/a | n/a | n/a | n/a | n/a |
| Not known | 18 | 0 | 0 | 0 | 88.9 | 11.1 | 11.1 | 5.6 | 0 | n/a | n/a | n/a | n/a | n/a |
| **Primary care-led investigation** | | | |  |  |  |  |  |  |  |  |  |  |  |
| Yes | 311 | 0 | 5.0 | 33.5 | 62.4 | 37.6 | 19.9 | 11.6 | 243 | 27.0 | 43.0 | 86.0 | 35.8 | 24.3 |
| No | 182 | 0 | 0 | 6.8 | 84.1 | 15.9 | 6.0 | 3.3 | 81 | 26.0 | 45.0 | 77.0 | 39.5 | 19.8 |
| Not known | 5 | 0 | 0 | 0 | 80.0 | 20.0 | 20.0 | 20.0 | 3 | 28.0 | 56.0 | 232.0 | 33.3 | 33.3 |

^a^The patient interval is defined as the number of days between the patient first observing signs or symptoms and the first presentation to a health professional.

^b^The referral to date informed interval is defined as the number of days from referral to the date the patient was informed whether they had cancer or not, both dates as recorded in the NCDA. Referral to date informed is only applicable for those on an urgent suspected cancer referral

Intervals are restricted to 0-730 days. Patients with a cancer diagnosed through screening are excluded.

^c^USC: Urgent Suspected Cancer

^d^Urgent referral not for suspected cancer

^e^MDC: Multi-disciplinary Diagnostic Centre

^f^RDC: Rapid Diagnostic Centre

^g^Emergency referral includes instances of patient self-referral

**Supplementary Table S6.** Number of consultations

| **Number of consultations** | **n (%)** |
| --- | --- |
| 0 | 153 |
| 1 | 277 (43.8) |
| 2 | 195 (30.8) |
| 3+ | 161 (25.4) |
| Not known | 133 |

**Supplementary Table S7.** Safety netting in NENs diagnosed in 2018

| **Safety netting** | **n (%)** |
| --- | --- |
| No | 438 (56.5) |
| Yes | 337 (43.5) |
| Not known | 144 |

**Supplementary Table S8.** Proportion and number of cases by symptom type for NCDA malignant NENs and NCDA all malignant cancers diagnosed in 2018. (95% Confidence intervals)

| **Symptom type** | **NCDA malignant NENs** | | **NCDA all malignant cancers** | |
| --- | --- | --- | --- | --- |
|  | **n** | **% (95% CI)** | **n** | **% (95% CI)** |
| Alarm symptom | 263 | 28.6 (25.7 - 31.7) | 20,820 | 32.5 (32.1 - 32.8) |
| Non-alarm symptom | 411 | 44.7 (41.5 - 48.0) | 26,291 | 41.0 (40.6 - 41.4) |
| Not applicable | 177 | 19.3 (16.8 - 22.0) | 10,844 | 16.9 (16.6 -17.2) |
| Not known | 68 | 7.4 (5.8 - 9.3) | 6,177 | 9.6 (9.4 - 9.9) |

**Supplementary Table S9.** Number of imaging investigations broken down by gender and cancer site

| **Cancer site** | **Male** | **Female** |
| --- | --- | --- |
| Bladder | 0 (0.0) | 2 (1.4) |
| Cancer of unknown primary | 17 (14.5) | 18 (12.2) |
| Colon | 8 (6.8) | 9 (6.1) |
| Lung | 46 (39.3) | 67 (45.3) |
| Oesophageal | 2 (1.7) | 1 (0.7) |
| Pancreatic | 11 (9.4) | 15 (10.1) |
| Rectal | 2 (1.7) | 1 (0.7) |
| Small intestine | 17 (14.5) | 12 (8.1) |
| Stomach | 4 (3.4) | 5 (3.4) |
| Thyroid | 4 (3.4) | 4 (2.7) |
| Other | 6 (5.1) | 14 (9.5) |
